# Rapid functional MRI in the mouse heart at 11.7T

**DOI:** 10.1186/1532-429X-15-S1-W31

**Published:** 2013-01-30

**Authors:** A Subgang, I Vernikouskaya, V Rasche

**Affiliations:** 1 CF-Small Animal MRI, Ulm University, Ulm, Germany

## Background

CINE imaging of the mouse heart is an established technique on high-field small animal MRI systems. The current imaging protocols normally comprise two-dimensional CINE imaging in 2- and 4-chamber geometry and a stack of short axis views for full functional coverage of the heart. For SNR constraints, acquisition times are still in the minute range for a single 2D slice, yielding rather long acquisition times for a complete short axis stack. Recently introduced cryogenically cooled resonators (CCR) offer the possibility of a significant increase in SNR, and hence offer the potential for rapid cine imaging of the mouse heart. The objective of this study was to investigate the feasibility of rapid functional imaging of the mouse heart utilizing the SNR gain provided by CCRs.

## Methods

Five wildtype mice (B6/C57) were enrolled in this study. In each mouse a comprehensive cardiac functional examination was performed on an 11.7T small animal system (Bruker BioSpec 117/16) with a 2-element cryogenic coil (CryoProbe, Bruker Biospin). The MR protocol was performed under isoflurane anesthesia (~1.5% concentration). Functional imaging was performed applying a self-gated acquisition/reconstruction approach (IntraGate, Bruker). In each mouse, cine data was acquired with different n=50, 100, 200, and 300 repetitions. Parallel imaging with an acceleration factor of 2 was used for all acquisitions. For further increasing the scan efficiency, all acquisitions were additionally performed with a half-scan factor of 1.6. Matrix size was 256x256, yielding 140 (parallel imaging) or 92 (parallel imaging plus halfscan) phase encoding steps. Overall acquisition times for a single slice resulted between 26.5s (50 reps, PI=2, HS=1.6) and 241.5s (300reps, PI=2, HS = 1). Imaging parameters were as: TE / TR = 0.95/5.75ms, flip angle = 20°, spatial resolution = 117x117x480µm3. From all data sets 20 cardiac phases were reconstructed and ejection fractions calculated semi-automatically (Segment).

## Results

The scans could be completed in all mice. The resulting image quality was sufficient for identification of the end-diastolic and end-systolic frame. Even though signal to noise (SNR) and apparent flow artifacts were clearly inferior in the 50 and 100 reps scans, no differences were observed in the calculated EF. The endocardial borders were clearly delineated from the blood pool, and the resulting endocardial contours did not show any substantial differences between the investigate protocols. The delineation of the epicardial contours was substantial impaired for the 50 and 100 reps acquisitions.

**Figure 1:**
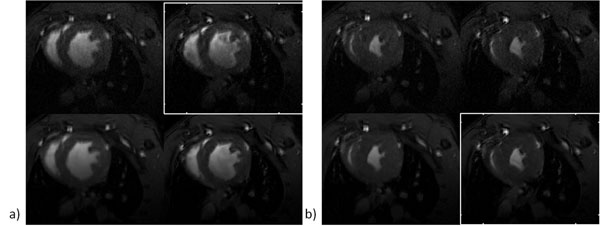


## Conclusions

Applying CCRs to functional heart imaging enables significantly shorter acquisition times. If the functional analysis is limited to EF analysis, decent image quality can already be obtained by a 25s scan. In case the epicardial borders are of interest, more repetitions are required for ensuring sufficient delineation of the epicaridal border.

